# Serum *Wisteria floribunda* agglutinin-positive Mac-2 binding protein in non-alcoholic fatty liver disease

**DOI:** 10.1371/journal.pone.0174982

**Published:** 2017-04-03

**Authors:** Lee-Lee Lai, Wah-Kheong Chan, Pavai Sthaneshwar, Nik Raihan Nik Mustapha, Khean-Lee Goh, Sanjiv Mahadeva

**Affiliations:** 1 Gastroenterology and Hepatology Unit, Department of Medicine, Faculty of Medicine, University of Malaya, Kuala Lumpur, Malaysia; 2 Clinical Diagnostic Laboratory, Department of Pathology, Faculty of Medicine, University of Malaya, Kuala Lumpur, Malaysia; 3 Department of Pathology, Hospital Sultanah Bahiyah, Alor Setar, Kedah, Malaysia; Medizinische Fakultat der RWTH Aachen, GERMANY

## Abstract

*Wisteria floribunda* agglutinin-positive Mac-2 binding protein (WFA^+^-M2BP) has been suggested to be useful for the assessment of disease severity in non-alcoholic fatty liver disease (NAFLD). Consecutive adult NAFLD patients who had a liver biopsy were included. Serum WFA^+^-M2BP level was measured using a lectin-antibody sandwich immunoassay using a chemiluminescence enzyme immunoassay machine (HISCL-5000, Sysmex, Kobe, Japan). The measured levels were indexed using the following equation: Cut-off index (COI) = ([WFA^+^-M2BP]_sample_−[WFA^+^-M2BP]_NC_) / ([WFA^+^-M2BP]_PC_−[WFA^+^-M2BP]_NC_), where PC = positive control and NC = negative control. Histopathological examination of liver biopsy specimen was reported according to Non-Alcoholic Steatohepatitis (NASH) Clinical Research Network Scoring System. Data for 220 cases were analyzed. The AUROC of the COI for the diagnosis of NASH was 0.65. The AUROC of the COI for the diagnosis of steatosis grade ≥2 and 3 was 0.64 and 0.53, respectively. The AUROC of the COI for the diagnosis of lobular inflammation grade ≥1, ≥2 and 3 was 0.57, 0.68 and 0.59, respectively. The AUROC of the COI for the diagnosis of hepatocyte ballooning grade ≥1 and 2 was 0.64 and 0.65, respectively. The AUROC of the COI for the diagnosis of fibrosis stage ≥1, ≥2, ≥3 and 4 was 0.61, 0.71, 0.74 and 0.84, respectively. Out of the 220 cases, 152 cases were the same 76 patients who had a repeat liver biopsy after 48 weeks of intervention. The AUROC of the change in the COI to detect improvement in steatosis, lobular inflammation, hepatocyte ballooning and fibrosis was 0.57, 0.54, 0.59 and 0.52, respectively. In conclusion, serum WFA^+^-M2BP was most useful for the diagnosis of significant fibrosis, advanced fibrosis and cirrhosis in NAFLD patients. However, it was less useful for differentiating NASH from non-NASH, and for diagnosis and follow-up of the individual histopathological components of NASH.

## Introduction

Non-alcoholic fatty liver disease (NAFLD) is the most common cause of chronic liver disease and is estimated to affect up to 30% of the general population [1]. Non-alcoholic steatohepatitis (NASH), the more severe form of NAFLD characterized by the presence of lobular inflammation and hepatocyte ballooning, could lead to fibrosis and cirrhosis, and is associated with an increased risk of liver-related complications and mortality [2]. The diagnosis of NASH is made by histopathological examination of a liver biopsy specimen, but a liver biopsy is invasive, associated with a small risk of serious complications [3], and poorly accepted by patients [4]. A simple and reliable non-invasive test is needed for the diagnosis and follow-up of patients with NASH. Recently, serum Mac-2 binding protein (M2BP) has been found to be significantly elevated in NASH patients compared with non-NASH patients, and has been suggested as a diagnostic tool for NASH [5]. Moreover, the advancement in glycol-chain biology has enabled the measurement of *Wisteria floribunda* agglutinin-positive Mac-2 binding protein (WFA^+^-M2BP) which is the isoform of the glycan structure of M2BP. Serum WFA+-M2BP level has been found to be increased according to the increasing severity of liver fibrosis in patients with chronic hepatitis [6]. Serum WFA^+^-M2BP has also been shown to correlate with the severity of liver fibrosis in NAFLD patients [7]. The aim of this study was to evaluate the usefulness of serum WFA+-M2BP for the diagnosis of NASH and fibrosis stage in NAFLD patients, and to compare it with routine biochemical markers and non-proprietary indices that are based on readily available parameters. This study also aimed to evaluate the usefulness of serum WFA^+^-M2BP in the follow-up of NASH patients after a period of intervention, which has never been reported before.

## Methods

This study was approved by the University of Malaya Medical Centre’s Medical Ethics Committee (Approval No.: 201401–0660) and conformed to the Declaration of Helsinki. All subjects provided written informed consent. The study subjects were NAFLD patients who had been screened for a clinical trial at the University of Malaya Medical Centre between 2012 and 2015. The diagnosis of NAFLD was based on ultrasonography finding of fatty liver and exclusion of significant alcohol intake, use of medications that can cause fatty liver, viral hepatitis B and C infection, and other causes of chronic liver disease where indicated [8]. Demographic, anthropometric and relevant clinical data were obtained using a standard protocol on the day of the liver biopsy procedure. Body mass index (BMI) was calculated by dividing weight in kilogram by the square of height in meters. Obesity was defined as BMI ≥ 25 kg per m^2^ [9]. Waist circumference was measured at the mid-point between the lowest margin of the least palpable rib and the top of the iliac crest in the standing position. Central obesity was defined as waist circumference > 90 cm for men and > 80 cm for women [10] Venous blood was drawn after an overnight fast on the day of the liver biopsy procedure for complete blood count, blood glucose, glycated haemoglobin (HbA1c), lipid profile, liver profile, and tests for viral hepatitis B and C infection. Additional blood sample was collected in a plain tube, processed to plasma and stored at -80°C until further analysis. Biochemical measurements were performed using standard laboratory procedures. The Elecsys HBsAg II assay and the Elecsys Anti-HCV II assay (Roche, Mannheim, Germany) were used to test for viral hepatitis B and C infection, respectively. Controls were recruited from persons attending the Endoscopy Unit, University of Malaya Medical Centre for investigation of dyspepsia or screening colonoscopy. All controls had no known medical illness and had an ultrasound examination to exclude fatty liver. Venous blood was drawn after an overnight fast, collected in a plain tube, processed to plasma and stored at -80°C until further analysis.

### Measurement of serum WFA^+^-M2BP and the Cut-Off Index (COI)

The serum WFA^+^-M2BP level was measured by a WFA-antibody sandwich immunoassay using a chemiluminescence enzyme immunoassay machine (HISCL-5000, Sysmex, Kobe, Japan), as previously reported [11]. The test was performed for all samples in a single session by a single investigator (PS). The measured levels were indexed using the following equation: cut-off index (COI) = ([WFA^+^-M2BP]_sample_−[WFA^+^-M2BP]_NC_) / ([WFA^+^-M2BP]_PC_−[WFA^+^-M2BP]_NC_), where PC = positive control and NC = negative control.

### Liver biopsy and histological assessment

Ultrasonography-guided percutaneous liver biopsy was performed by either one of two experienced operators (WKC, SM) using 18G Temno^®^ II semi-automatic biopsy needle (Cardinal Health, Dublin, Ohio, USA). Liver biopsy specimens were processed using standard laboratory procedures. Liver biopsy slides were stained with hematoxylin and eosin stain and masson trichrome stain. Liver biopsy slides were examined by an experienced histopathologist (NRNM) who was blinded to clinical data. NASH was defined as the presence of steatosis, lobular inflammation and ballooning with or without fibrosis. Histopathological findings were reported according to the Non-Alcoholic Steatohepatitis Clinical Research Network Scoring System [12]. Fibrosis stages 1a, 1b and 1c were considered stage 1 for the purpose of analysis.

### Liver stiffness measurement

Liver stiffness measurement (LSM) was performed after overnight fasting using the Fibroscan 502 Touch with the M probe (EchoSens, Paris, France) on the same day as the liver biopsy procedure. Adequate pressure of the probe on the skin surface, good layering on TM mode and a straight imaginary line on A mode were ensured for each measurement. An examination was considered successful if there were ten valid measurements, and reliable if the interquartile range (IQR)/median for LSM was ≤ 30%, or the LSM was < 7.1 kPa when the IQR/median for LSM was > 30% [13]. Subjects with unsuccessful or unreliable examination were excluded from the analysis on LSM.

### Statistical analysis

Data were analysed using a standard statistical software program (SPSS 15.0). Continuous variables were expressed as mean ± standard deviation or median (interquartile range) and analyzed using t-test, Mann-Whitney test or Kruskal-Wallis test, as appropriate. Categorical variables were expressed as percentages and analyzed using chi-square test or Fisher’s exact test, as appropriate. Significance was assumed when p < 0.05. Boxplots were used to compare the COI of healthy controls, non-NASH patients and NASH patients, the different grades of steatosis, lobular inflammation and ballooning, and the different fibrosis stages. The performance of the COI for the diagnosis of NASH, the different grades of steatosis, lobular inflammation and hepatocyte ballooning, and the different stages of fibrosis were determined using area under receiver-operating characteristics curve (AUROC). AUROC was interpreted as follows: 0.90–1.00 = excellent, 0.80–0.90 = good, 0.70–0.80 = fair, < 0.70 = poor. Optimal cut-off values for the COI were the values that provided the greatest sum of sensitivity and specificity. The sensitivity, specificity, positive predictive value and negative predictive value using the optimal cut-off values were determined. Using the AUROC, the performance of the COI for the diagnosis of NASH was compared with serum alanine aminotransferase (ALT), aspartate aminotransferase (AST) and gamma glutamyl transpeptidase (GGT) levels. Similarly, the performance of the COI for the diagnosis of fibrosis stages was compared with the AST to platelet ratio index (APRI) [14], fibrosis-4 index (FIB-4) [15] and NAFLD fibrosis score [16]. Similarly, the performance of the change in the COI and the percentage of change in the COI for detection of improvement in liver histology after a period of intervention was evaluated using AUROCs.

## Results

### Patient characteristics

Data for 220 liver biopsies were analyzed. The data can be found in Supporting Information S1 File. Patient characteristics are summarized in Tables 1 and 2. The mean age of the study population was 50.1 ± 11.5 years, and consisted of 51.8% men. Median (IQR) length of liver biopsy specimen was 15 (12–16) mm, and number of portal tracts was 8 (6–10). The study population consisted of 72.7% NASH patients. The distribution of fibrosis stages is as follows: F0, 32.3%; F1, 40.9%; F2, 7.3%; F3, 16.4%; F4, 3.2%. Patients with NASH were older, more likely to be female, had greater BMI and waist circumference, and were more likely to have central obesity, diabetes mellitus and hypertension compared with non-NASH patients. Patients with NASH also had higher serum FBS, HbA1c, ALT, AST and GGT levels, and lower serum TC and LDL levels. Thirty-eight healthy controls were included in this study. The mean age of the healthy controls was 35.2 ± 17.1 years old, and consisted of 18.4% men. Their mean BMI and waist circumference was 21.6 ± 3.0 kg per m^2^ and 74.6 ± 9.3 cm, respectively, and obesity and central obesity was present in 13.2% and 18.4%, respectively.

**Table 1:** Patient characteristics.

	Overall, n = 220	Non-NASH patients, n = 60	NASH patients, n = 160	p
Age, years	50.1 ± 11.5	47.3 ± 10.9	51.2 ± 11.5	0.024
Male, %	51.8	62.5	47.5	0.036
BMI, kg per m^2^	29.9 ± 4.4	28.8 ± 4.1	30.4 ± 4.4	0.014
Obesity, %	87.7	81.2	88.8	0.450
Waist circumference, cm	98.7 ± 10.3	95.5 ± 9.4	99.8 ± 10.4	0.006
Central obesity, %	94.5	90.6	98.8	0.002
Diabetes mellitus, %	52.7	34.4	60.6	0.000
Hypertension, %	60.0	43.8	66.9	0.001
Dyslipidemia, %	74.5	70.3	76.9	0.195
FBS, mmol/L	5.9 (5.1–7.2)	5.5 (5.0–6.2)	6.0 (5.2–7.4)	0.021
HbA1c, %	6.2 (5.6–7.3)	5.7 (5.3–6.1)	6.6 (5.7–7.6)	0.000
TG, mmol/L	1.60 (1.30–2.00)	1.60 (1.30–2.00)	1.6 (1.3–2.0)	0.803
TC, mmol/L	4.80 (4.20–5.60)	5.10 (4.40–5.80)	4.65 (4.10–5.48)	0.016
HDL, mmol/L	1.13 (0.99–1.33)	1.20 (1.00–1.40)	1.11 (0.97–1.30)	0.121
LDL, mmol/L	2.84 (2.31–3.57)	3.03 (2.48–3.57)	2.71 (2.22–3.57)	0.048
ALT, IU/L	65 (44–102)	49 (35–73)	72 (48–111)	0.000
AST, IU/L	40 (29–61)	31 (23–39)	49 (32–71)	0.000
GGT, IU/L	79 (41–123)	45 (31–91)	83 (50–130)	0.000

NASH was defined as the presence of steatosis, lobular inflammation and ballooning with or without fibrosis.

p were calculated using independent t-test or Mann-Whitney test, where appropriate, for continuous variables, and chi-square test or Fisher exact test, where appropriate, for categorical variables.

BMI, body mass index; FBS, fasting blood sugar; HbA1c, glycated hemoglobin; TG, triglyceride; TC, total cholesterol; HDL, high-density lipoprotein cholesterol; LDL; low-density lipoprotein cholesterol; ALT, alanine aminotransferase; AST, aspartate aminotransferase; GGT, gamma glutamyl transpeptidase

**Table 2:** Characteristics of patients according to fibrosis stages.

	F0, n = 71	F1, n = 90	F2, n = 16	F3, n = 36	F4, n = 7
Age, years	46.4 ± 10.3	50.2 ± 12.4	48.3 ± 12.3	56.5 ± 7.9	58.3 ± 7.9
Male, %	52.1	51.1	68.8	47.2	42.9
BMI, kg per m^2^	29.2 ± 4.0	30.1 ± 4.3	30.7 ± 3.9	30.6 ± 5.6	30.2 ± 3.6
Obesity, %	85.9	85.6	100	88.9	100
Waist circumference, cm	97.0 ± 10.0	97.4 ± 9.4	103.1 ± 9.9	102.9 ± 12.5	99.5 ± 7.2
Central obesity, %	90.1	94.4	100	100	100
Diabetes mellitus, %	26.8	57.8	43.8	88.9	85.7
Hypertension, %	47.9	57.8	56.2	88.9	71.4
Dyslipidemia, %	69.0	76.7	68.8	83.3	71.4
FBS, mmol/L	5.4 (4.9–5.9)	6.0 (5.0–7.2)	6.0 (5.1–7.3)	7.3 (5.6–9.0)	6.8 (6.2–7.1)
HbA1c, %	5.7 (5.4–6.5)	6.3 (5.7–7.4)	6.0 (5.4–7.3)	7.2 (6.3–9.3)	6.5 (5.4–7.2)
TG, mmol/L	1.55 (1.18–2.00)	1.60 (1.30–2.10)	1.45 (1.05–1.85)	1.65 (1.30–1.98)	1.30 (0.90–2.10)
TC, mmol/L	4.90 (4.38–5.70)	4.75 (4.10–5.80)	4.65 (4.00–5.38)	4.40 (3.83–5.08)	4.30 (4.10–4.90)
HDL, mmol/L	1.20 (0.99–1.40)	1.11 (0.98–1.27)	1.06 (0.85–1.27)	1.15 (1.00–1.30)	1.26 (1.16–1.60)
LDL, mmol/L	2.95 (2.49–3.62)	2.91 (2.23–3.69)	3.15 (2.31–3.61)	2.43 (1.95–3.48)	2.41 (1.73–2.66)
ALT, IU/L	55 (41–79)	66 (42–108)	98 (71–121)	72 (47–125)	56 (44–101)
AST, IU/L	33 (25–40)	43 (29–60)	60 (30–71)	63 (43–86)	53 (36–72)
GGT, IU/L	48 (33–96)	71 (40–109)	98 (56–184)	115 (79–205)	123 (97–171)

BMI, body mass index; FBS, fasting blood sugar; HbA1c, glycated hemoglobin; TG, triglyceride; TC, total cholesterol; HDL, high-density lipoprotein cholesterol; LDL; low-density lipoprotein cholesterol; ALT, alanine aminotransferase; AST, aspartate aminotransferase; GGT, gamma glutamyl transpeptidase

### COI in healthy controls, non-NASH patients and NASH patients

**Fig 1:**
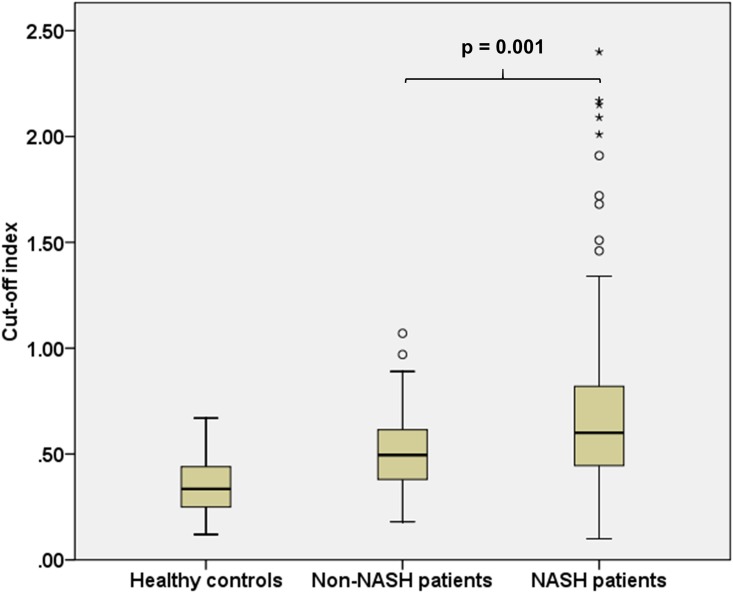
Boxplot showing the cut-off index in healthy controls, non-NASH patients and NASH patients. NASH was defined as the presence of steatosis, lobular inflammation and ballooning with or without fibrosis. p value was calculated using Mann-Whitney test.

The COI in healthy controls, non-NASH patients and NASH patients are shown in Fig 1. The median (IQR) for COIs for healthy controls, non-NASH patients and NASH patients was 0.34 (0.25–0.44), 0.50 (0.38–0.62) and 0.60 (0.44–0.82), respectively. The COIs were significantly higher in NAFLD patients compared with healthy controls (p < 0.001), and in NASH patients compared with non-NASH patients (p = 0.001).

### COI for the diagnosis of NASH

**Fig 2:**
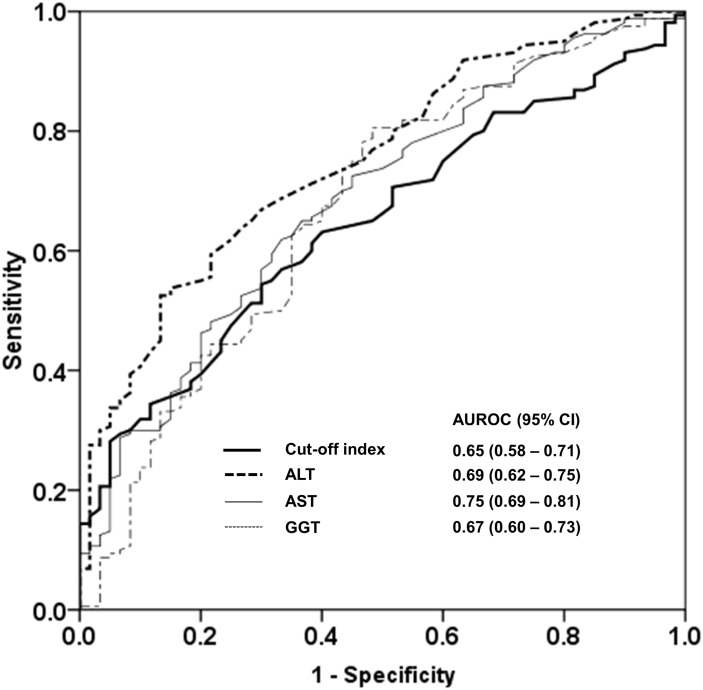
A comparison of the cut-off index with serum ALT, AST and GGT levels for differentiating NASH from non-NASH among NAFLD patients. NASH was defined as the presence of steatosis, lobular inflammation and ballooning with or without fibrosis. AUROC was interpreted as follows: 0.90–1.00 = excellent, 0.80–0.90 = good, 0.70–0.80 = fair, 0.70 = poor.

The AUROC of the COI for differentiating NASH from non-NASH among NAFLD patients was 0.65. Using the optimal cut-off of 0.58, the sensitivity, specificity, positive predictive value and negative predictive value of the COI for differentiating NASH from non-NASH among NAFLD patients was 54.4%, 70%, 82.9% and 36.5%, respectively. A comparison of the COI with serum ALT, AST and GGT levels for differentiating NASH from non-NASH among NAFLD patients is shown in Fig 2.

### COI for steatosis, lobular inflammation and hepatocyte ballooning

The COI according to grades of steatosis is shown in S1 Fig. The median (IQR) for COI for steatosis grades 1, 2 and 3 was 0.64 (0.50–0.97), 0.53 (0.41–0.66) and 0.54 (0.37–0.76), respectively (p = 0.007). The COI was significantly higher in patients with steatosis grade 1 compared with steatosis grade 2 (p = 0.001), but not significantly different between patients with steatosis grade 2 compared with steatosis grade 3 (p = 0.693). The AUROC for the COI for diagnosis of steatosis grade ≥ 2 and 3 was 0.64 and 0.53, respectively.

The COI according to grades of lobular inflammation is shown in S2 Fig. The median (IQR) for COI for lobular inflammation grades 0, 1, 2 and 3 was 0.55 (0.38–0.64), 0.51 (0.39–0.64), 0.65 (0.49–0.87) and 0.83 (0.36–1.30), respectively (p < 0.001). The COI was significantly higher in patients with lobular inflammation grade 2 compared with lobular inflammation grade 1 (p < 0.001), but not significantly different between patients with lobular inflammation grade 0 compared with lobular inflammation grade 1 (p = 0.826), and between patients with lobular inflammation grade 2 compared with lobular inflammation grade 3 (p = 0.839). The AUROC for the COI for diagnosis of lobular inflammation grade ≥ 1, ≥ 2 and 3 was 0.57, 0.68 and 0.59, respectively.

The COI according to grades of hepatocyte ballooning is shown in S3 Fig. The median (IQR) for COI for hepatocyte ballooning grades 0, 1 and 2 was 0.50 (0.38–0.62), 0.58 (0.44–0.74) and 0.69 (0.48–1.04), respectively (p < 0.001). The COI was significantly higher in patients with hepatocyte ballooning grade 1 compared with hepatocyte ballooning grade 0 (p = 0.021), and in patients with hepatocyte ballooning grade 2 compared with hepatocyte ballooning grade 1 (p = 0.020). The AUROC for the COI for diagnosis of hepatocyte ballooning grade ≥ 1 and 2 was 0.64 and 0.65, respectively.

### COI according to fibrosis stages

**Fig 3:**
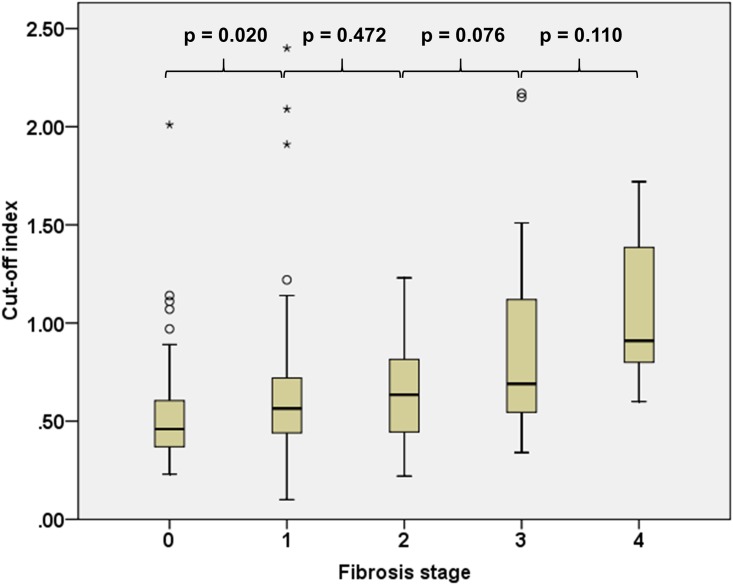
Boxplot showing the cut-off index according to fibrosis stages (p < 0.001 across groups). p value across groups was calculated using Kruskal-Wallis test and between groups was calculated using Mann-Whitney test.

The COI according to fibrosis stages is shown in Fig 3. The median (IQR) for COI for fibrosis stages F0, F1, F2, F3 and F4 was 0.46 (0.37–0.61), 0.57 (0.44–0.72), 0.64 (0.43–0.82), 0.69 (0.54–1.14) and 0.91 (0.70–1.68), respectively (p < 0.001). The COI was significantly higher in patients with fibrosis stage F1 compared with fibrosis stage F0 (p = 0.020), but not significantly different between patients with fibrosis stage F1 compared with fibrosis stage F2 (p = 0.472), fibrosis stage F2 compared with fibrosis stage F3 (p = 0.076), and fibrosis stage F3 compared with fibrosis stage F4 (p = 0.110).

### COI for the diagnosis of fibrosis stages

**Fig 4:**
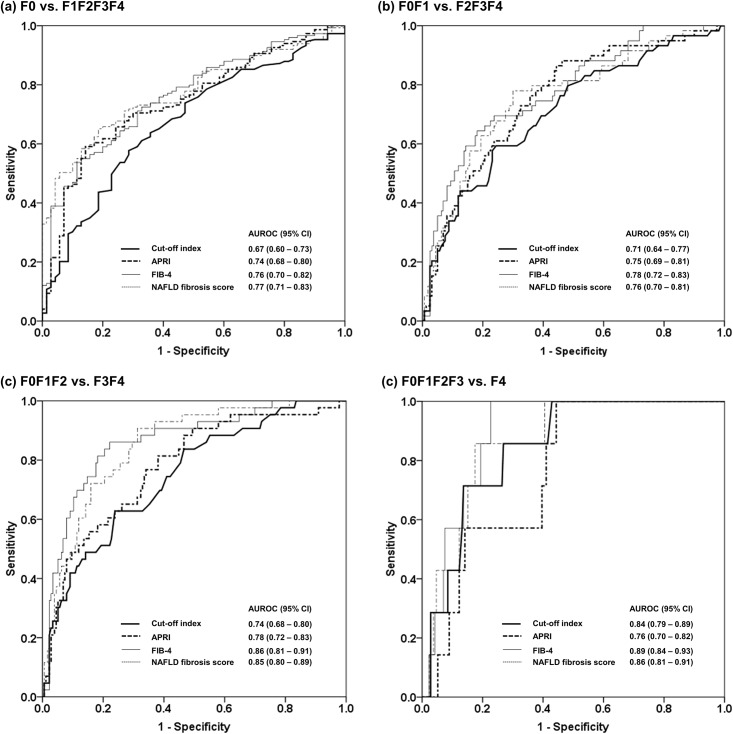
A comparison of the cut-off index with FIB-4, APRI and the NAFLD fibrosis score for the diagnosis of fibrosis stages. AUROC was interpreted as follows: 0.90–1.00 = excellent, 0.80–0.90 = good, 0.70–0.80 = fair, 0.70 = poor.

The AUROC, optimal cut-off, sensitivity, specificity, positive predictive value and negative predictive value of the COI for the diagnosis of fibrosis stages is shown in Table 3. The COI was good for F0F1F2F3 vs. F4, and fair for F0F1F2 vs. F3F4 and F0F1 vs. F2F3F4, but poor for F0 vs. F1F2F3F4. A comparison of the COI with the APRI, FIB-4 and NAFLD fibrosis score for the diagnosis of fibrosis stages is shown in Fig 4. The FIB-4 and NAFLD fibrosis score were better than the COI for F0F1F2 vs. F3F4. Fibroscan was successful and reliable in 213 cases. The AUROC of liver stiffness measurement using liver stiffness measurement for diagnosis of fibrosis stage ≥ F1, ≥ F2, ≥ F3 and F4 was 0.82, 0.88, 0.93 and 0.92, respectively.

**Table 3:** The AUROC, optimal cut-off, sensitivity, specificity, positive predictive value and negative predictive value of the cut-off index for the diagnosis of fibrosis stages.

	AUROC (95% CI)	Optimal cut-off	Sensitivity, %	Specificity, %	Positive predictive value, %	Negative predictive value, %
≥F1	0.67 (0.60–0.73)	0.57	57.7	70.4	80.4	44.2
≥F2	0.71 (0.64–0.77)	0.66	59.3	75.2	46.7	83.4
≥F3	0.74 (0.68–0.80)	0.69	62.8	75.7	38.6	89.3
F4	0.84 (0.79–0.89)	0.70	85.7	72.8	9.4	99.4

AUROC was interpreted as follows: 0.90–1.00 = excellent, 0.80–0.90 = good, 0.70–0.80 = fair, 0.70 = poor.

Optimal cut-off was the cut-off with highest sum of sensitivity and specificity.

Exploratory analyses were performed to look into the combination of the COI and the FIB-4 and NAFLD fibrosis score for the diagnosis of advanced fibrosis, and the combination of the COI and the APRI for the diagnosis of cirrhosis. The cut-off for COI to exclude advanced fibrosis was selected with at least 90% sensitivity and the highest possible specificity, while the cut-off for COI to diagnose advanced fibrosis was selected for at least 90% specificity and the highest possible sensitivity. The cut-offs were 0.45 and 0.90, respectively. These cut-offs provided negative predictive value of 93.8% (60/64) and positive predictive value of 48.4% (16/33) for advanced fibrosis. A total of 55.9% (123/220) of patients had values between 0.45 and 0.90 and were considered to be in the indeterminate group.

Using accepted cut-offs of 1.45 and 3.25, FIB-4 had a negative predictive value of 91.8% (156/170) and a positive predictive value of 33.3% (2/6) for advanced fibrosis. A total of 19.6% (43/219) of patients had values between 1.45 and 3.25 and were considered to be in the indeterminate group. Combining the COI and FIB-4 in all patients increased the negative predictive value to 96.5% (55/57) and positive predictive value to 50% (2/4) but resulted in 72.1% (158/219) of patients having discordant or indeterminate results. If COI was only performed for patients with indeterminate FIB-4, the negative predictive value was 91.0% (161/177), the positive predictive value was 68.2% (15/22), and the percentage of patients with indeterminate results was reduced to 9.1% (20/219).

Using accepted cut-offs of -1.455 and 0.676, the NAFLD fibrosis score had a negative predictive value of 92.6% (137/148) and a positive predictive value of 71.4% (5/7) for advanced fibrosis. A total of 29.2% (64/219) of patients had values between -1.455 and 0.676 and were considered to be in the indeterminate group. Combining COI and NAFLD fibrosis score increased the negative predictive value to 96.2% (50/52) and positive predictive value to 100% (5/5) but resulted in 74.0% (162/219) of patients to have discordant or indeterminate results. If COI was only performed for patients with indeterminate NAFLD fibrosis score, the negative predictive value was 91.9% (147/160), the positive predictive value was 60.9% (14/23), and the percentage of patients with indeterminate results was reduced to 16.4% (36/219).

The cut-off for COI to exclude cirrhosis was selected with at least 90% sensitivity and the highest possible specificity, while the cut-off for COI to diagnose cirrhosis was selected for at least 90% specificity and the highest possible sensitivity. The cut-offs were 0.60 and 1.06, respectively. These cut-offs provided negative predictive value of 100% (121/121) and positive predictive value of 12.5% (3/24) for cirrhosis. A total of 34.1% (75/220) of patients had values between 0.60 and 1.06 and were considered to be in the indeterminate group.

Using accepted cut-offs of 0.5 and 1.5, APRI had a negative predictive value of 99.2% (123/124) and a positive predictive value of 0% (0/7) for cirrhosis. A total of 40.2% (88/219) of patients had values between 0.5 and 1.5 and were considered to be in the indeterminate group. Combining COI and APRI in all patients resulted in a negative predictive value of 0% (0/86) and positive predictive value of 0% (0/3). A total of 59.4% (130/219) of patients had discordant or indeterminate results. If COI was only performed for patients with indeterminate APRI, the negative predictive value was 99.4% (156/157), the positive predictive value was 12.0% (3/25), and the percentage of patients with indeterminate results was reduced to 16.9% (37/219).

### Changes in the COI to detect improvement in histology

**Fig 5:**
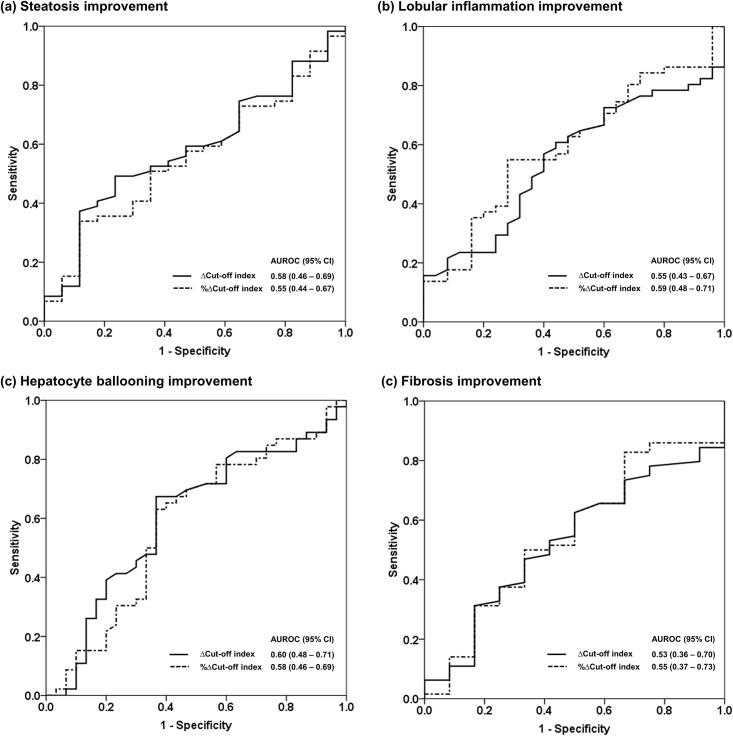
Change in cut-off index and percentage of change in cut-off index for detection of improvement in steatosis, lobular inflammation, hepatocyte ballooning and fibrosis. AUROC was interpreted as follows: 0.90–1.00 = excellent, 0.80–0.90 = good, 0.70–0.80 = fair, 0.70 = poor.

Out of the 220 liver biopsies, 152 were paired liver biopsies from 76 patients who had a repeat liver biopsy after 48 weeks of intervention. The AUROC of the change in the COI to detect improvement in steatosis, lobular inflammation, hepatocyte ballooning and fibrosis was 0.58, 0.55, 0.60 and 0.53, respectively. The AUROC of the percentage of change in the COI to detect improvement in steatosis, lobular inflammation, hepatocyte ballooning and fibrosis was 0.55, 0.59, 0.58 and 0.55, respectively (Fig 5).

## Discussion

Fucosylated glycoproteins produced in the liver are normally secreted into bile but not into the bloodstream. It has been postulated that the fucosylation-based sorting mechanism is disrupted in ballooned hepatocytes leading to secretion of the fucosylated glycoproteins into the bloodstream. Mac-2bp is one of such glycoproteins, and its level in the bloodstream has been shown to be significantly increased in NASH patients compared with non-NASH patients. In a study on 127 biopsy-proven NAFLD patients, NASH patients had significantly higher serum WFA^+^-M2BP levels compared with non-NASH patients, and serum WFA^+^-M2BP level was found to be good for differentiating NASH patients from non-NASH patients with an AUROC of 0.84 [5]. A subsequent study on serum M2BP levels in 289 biopsy-proven NAFLD patients focused on its utility in diagnosing fibrosis stage and provided no information on its accuracy for differentiating NASH patients from non-NASH patients [7]. In another study on 134 biopsy-proven NASH patients with a NAS of ≥ 5, significant overlap was observed in the serum M2BP level of patients with different grades of steatosis and lobular inflammation. Although serum M2BP levels were significantly higher in patients with grade 2 compared with grade 1 hepatocyte ballooning, the AUROC of serum M2BP level for the diagnosis of the higher grade of hepatocyte ballooning was not reported [17].

In this study, we found serum WFA^+^-M2BP levels to be poor for the diagnosis of steatosis, lobular inflammation, hepatocyte ballooning and NASH in NAFLD patients, with AUROCs of < 0.70. However, the performance of the test was fair to good for detection of significant fibrosis (≥ F2), advanced fibrosis (≥ F3) and cirrhosis (F4), and was comparable to popular indices such as APRI, FIB-4 and the NAFLD fibrosis score. These results seem reasonable because WFA^+^-M2BP has been developed as a marker for the diagnosis of liver fibrosis. The results are also somewhat consistent with previous reports [5, 7, 16], although the FIB-4 and NAFLD fibrosis score performed better than the serum WFA^+^-M2BP level for advanced fibrosis in our study.

The performance of serum WFA^+^-M2BP level for the diagnosis of the different stages of fibrosis was lower in our study when compared with an earlier study [7]. The serum WFA^+^-M2BP level in cirrhotic patients is numerically much higher than lower stages of fibrosis. We believe the serum WFA^+^-M2BP level will be even higher in cirrhotic patients with more advanced liver disease. Hence, we hypothesize that the AUROCs of serum WFA^+^-M2BP level for the diagnosis of the different stages of fibrosis may be increased (1) when there is a larger proportion of cirrhotic patients, and (2) when there is a larger proportion of cirrhotic patients with more advanced liver disease in the study population, vice versa. The earlier study did have a larger proportion of cirrhotic patients compared with our study, 17.6% vs. 3.2%. The indication for liver biopsy was not clearly described in the earlier study. On the other hand, our study population consisted of NAFLD patients who underwent a liver biopsy as part of screening for a clinical trial, and these were consecutive NAFLD patients seen in our clinic, who were suspected to have NASH. Moreover, NAFLD patients who obviously had cirrhosis based on biochemical or radiological findings were excluded. Thus, we believe our study population would reflect the NAFLD patients who would benefit from a serum biomarker for assessing the severity of their disease in day-to-day clinical practice.

The combination of COI and the FIB-4 and NAFLD fibrosis score in all patients for the diagnosis of advanced fibrosis improved the negative and positive predictive values but resulted in a high rate of discordant or indeterminate results. Using the COI for patients with high FIB-4 and NAFLD fibrosis score reduced the proportion of patients in the indeterminate group without significant effect on the negative and positive predictive values, and seemed feasible. The combination of COI and the APRI in all patients for the diagnosis of cirrhosis was useless. However, using the COI for patients with indeterminate APRI reduced the proportion of patients in the indeterminate group without significant effect on the negative and positive predictive values, and seemed feasible. These findings on the combination of COI and the FIB-4, NAFLD fibrosis score and APRI deserve further study and validation.

This is the first report on the use of serum WFA^+^-M2BP level for the detection of improvement in liver histology following intervention. Unfortunately, serum WFA^+^-M2BP level did not appear useful for this purpose with AUROCs of < 0.70 for the detection of improvement in steatosis, lobular inflammation, hepatocyte ballooning and fibrosis. The search for a simple and reliable non-invasive marker for the diagnosis and follow-up of NAFLD patients therefore remains elusive.

The data for this study was collected prospectively and the blood sample was collected on the same day of the liver biopsy procedure to minimize differences due to changes over time. Despite our best effort, this study had several limitations. Firstly, study subjects were from a tertiary care hospital who had more severe liver disease compared with NAFLD patients in the general population. Thus, the findings of this study may not be generalizable to NAFLD patients in the general population. Secondly, despite being the current best standard, the use of a liver biopsy specimen as the reference is inherently subjected to sampling and observer variability. Thirdly, the absence of fatty liver in healthy controls was based on ultrasonography, which may not detect mild hepatic steatosis. However, performing a liver biopsy in healthy controls to exclude fatty liver for the purpose of research is not ethical.

In conclusion, serum WFA^+^-M2BP level was found to be fair to good for the diagnosis of significant fibrosis, advanced fibrosis and cirrhosis in NAFLD patients, but poor for differentiating NASH from non-NASH. It was also found to be poor for the diagnosis of the grade of steatosis, lobular inflammation and hepatocyte ballooning, and for the follow-up for improvement in liver histology following intervention.

## Supporting information

S1 Fig Boxplot showing the cut-off index according to steatosis grades (p = 0.007 across groups). p value across groups was calculated using Kruskal-Wallis test and between groups was calculated using Mann-Whitney test.
(TIF)

S2 Fig Boxplot showing the cut-off index according to lobular inflammation grades (p < 0.001 across groups). p value across groups was calculated using Kruskal-Wallis test and between groups was calculated using Mann-Whitney test.
(TIF)

S3 Fig Boxplot showing the cut-off index according to hepatocyte ballooning grades (p < 0.001 across groups). p value across groups was calculated using Kruskal-Wallis test and between groups was calculated using Mann-Whitney test.
(TIF)

S1 File Data underlying the results presented in this paper. (SAV)
