# Supplementary figures and images for: Serum Wisteria floribunda agglutinin-positive Mac-2 binding protein in non-alcoholic fatty liver disease
Source: PLoS One. 2017 Apr 3;12(4):e0174982. doi: 10.1371/journal.pone.0174982 (PMC5378406; doi:10.1371/journal.pone.0174982)

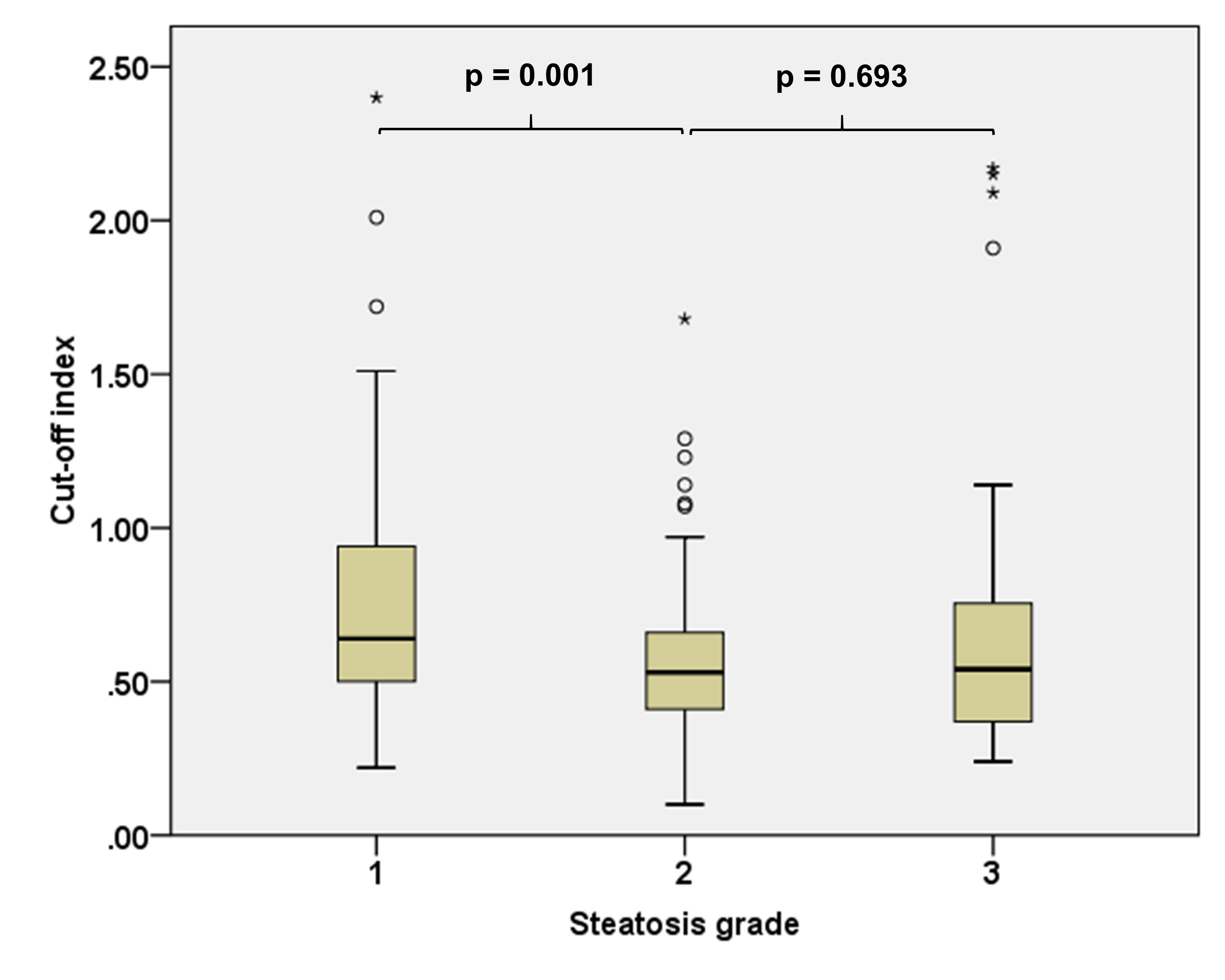

Supplement: S1 Fig — p value across groups was calculated using Kruskal-Wallis test and between groups was calculated using Mann-Whitney test. (TIF) [file pone.0174982.s001.tif]

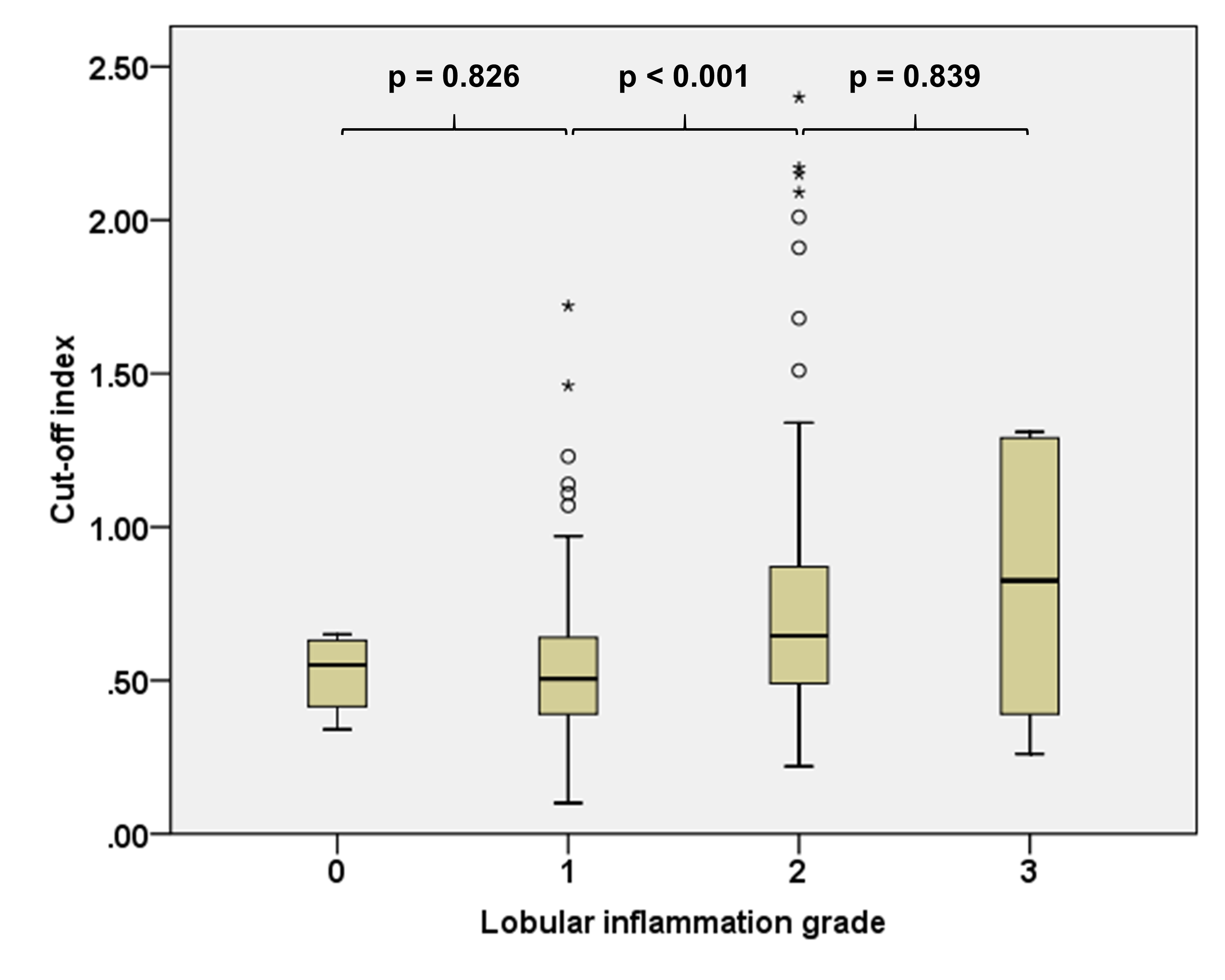

Supplement: S2 Fig — p value across groups was calculated using Kruskal-Wallis test and between groups was calculated using Mann-Whitney test. (TIF) [file pone.0174982.s002.tif]

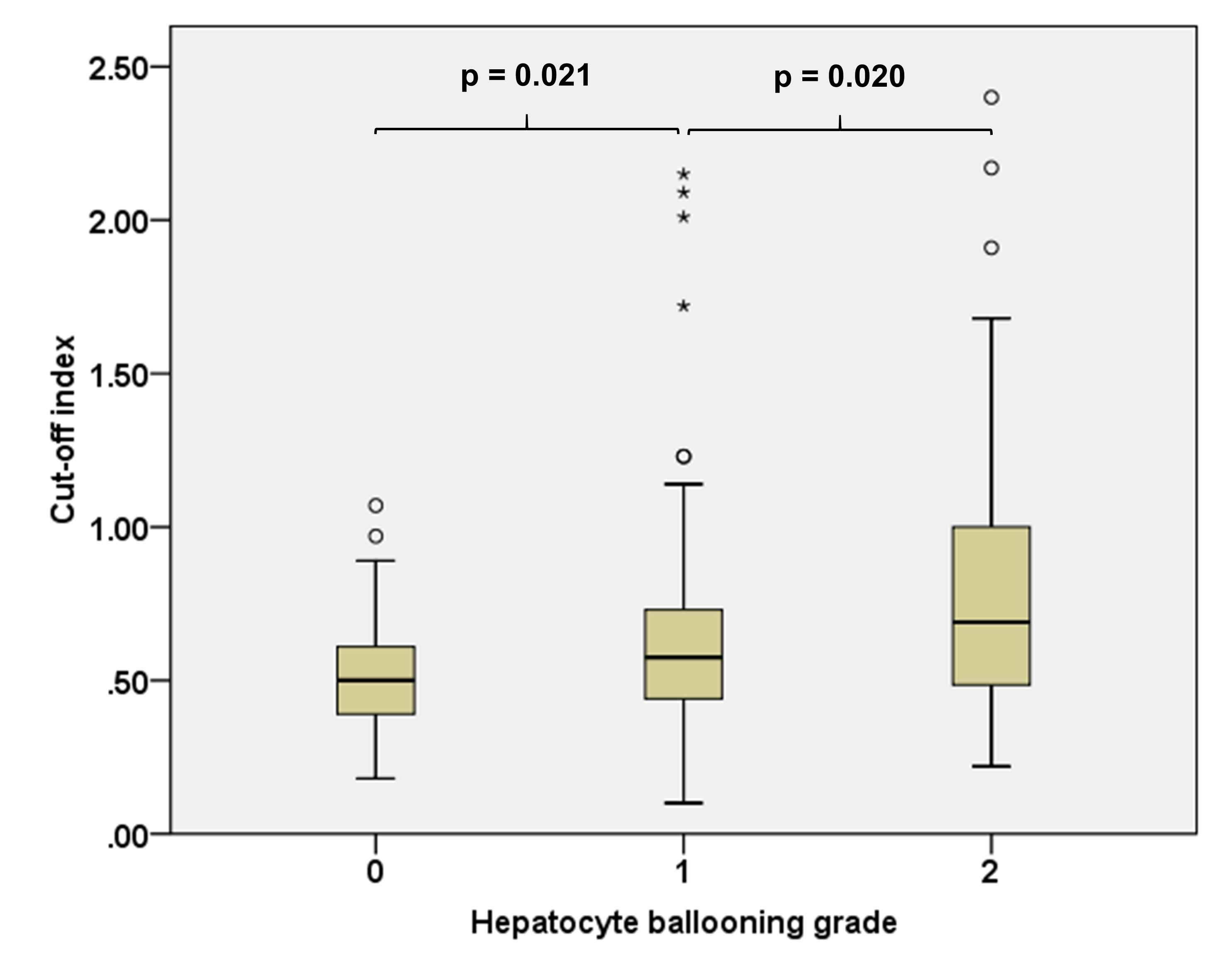

Supplement: S3 Fig — p value across groups was calculated using Kruskal-Wallis test and between groups was calculated using Mann-Whitney test. (TIF) [file pone.0174982.s003.tif]
